# Riboflavin Reduces Pro-Inflammatory Activation of Adipocyte-Macrophage Co-culture. Potential Application of Vitamin B2 Enrichment for Attenuation of Insulin Resistance and Metabolic Syndrome Development

**DOI:** 10.3390/molecules21121724

**Published:** 2016-12-15

**Authors:** Agnieszka Irena Mazur-Bialy, Ewa Pocheć

**Affiliations:** 1 Department of Ergonomics and Exercise Physiology, Faculty of Health Science, Jagiellonian University Medical College, Grzegorzecka 20, 31-531 Krakow, Poland; 2 Department of Glycoconjugate Biochemistry, Institute of Zoology, Jagiellonian University, Gronostajowa 9, 30-387 Krakow, Poland; ewa.pochec@uj.edu.pl

**Keywords:** riboflavin, adipocyte tissue, inflammation, obesity, insulin resistance, metabolic syndrome, obesity-related inflammation

## Abstract

Due to the progressive increase in the incidence of obese and overweight individuals, cardiometabolic syndrome has become a worldwide pandemic in recent years. Given the immunomodulatory properties of riboflavin, the current study was performed to investigate the potency of riboflavin in reducing obesity-related inflammation, which is the main cause of insulin resistance, diabetes mellitus 2 or arteriosclerosis. We determined whether pretreatment with a low dose of riboflavin (10.4–1000 nM) affected the pro-inflammatory activity of adipocyte-macrophage co-culture (3T3 L1-RAW 264.7) following lipopolysaccharide stimulation (LPS; 100 ng/mL) which mimics obesity-related inflammation. The apoptosis of adipocytes and macrophages as well as tumor necrosis factor-alpha (TNF-α), interleukin 6 (IL-6), interleukin 1beta (IL-1β), monocyte chemotactic protein 1 (MCP-1), high-mobility group box 1 (HMGB1), transforming growth factor–beta 1 (TGFβ), interleukin 10 (IL-10), inducible nitric oxide synthase (iNOS), nitric oxide (NO), matrix metalloproteinase 9 (MMP-9), tissue inhibitor of metalloproteinases-1 (TIMP-1) expression and release, macrophage migration and adipokines (adiponectin and leptin) were determined. Our results indicated an efficient reduction in pro-inflammatory factors (TNFα, IL-6, MCP-1, HMGB1) upon culture with riboflavin supplementation (500–1000 nM), accompanied by elevation in anti-inflammatory adiponectin and IL-10. Moreover, macrophage migration was reduced by the attenuation of chemotactic MCP-1 release and degradation of the extracellular matrix by MMP-9. In conclusion, riboflavin effectively inhibits the pro-inflammatory activity of adipocyte and macrophage co-cultures, and therefore we can assume that its supplementation may reduce the likelihood of conditions associated with the mild inflammation linked to obesity.

## 1. Introduction

Due to the progressive increase in the incidence of obese and overweight individuals, especially in developing countries, cardiometabolic syndrome has become a worldwide pandemic in recent years [1]. Metabolic syndromes are associated with the presence of macrophages with classically activated phenotypes in adipose tissue [2,3]. The interaction between adipocytes and adipose tissue–infiltrating macrophages contributes to insulin resistance in tissues, dyslipidemia, diabetes and cardiovascular syndrome, which are the results of obesity [4].

Previous studies have clearly demonstrated that functions of adipose tissue are not only limited to metabolic and endocrine activities, but adipocytes also show some features typical of immune cells [4,5,6]. Obesity-related enlargement of adipocytes is associated with an increase in free fatty acids (FFA) release and the production of several pro-inflammatory factors [7], including tumor necrosis factor-alpha (TNF-α) [8,9], interleukin 6 (IL-6), monocyte chemotactic protein 1 (MCP-1), inducible nitric oxide synthase (iNOS), and transforming growth factor–beta 1 (TGF-β1) [7,10,11]. Both of these activities result in adipocyte pro-inflammatory responses and promote the development of insulin resistance in adipose tissue [12,13].

Riboflavin (vitamin B2) belongs to the group of natural compounds present in the human diet and is an essential factor for the proper functioning of the immune system [14]. Searching among natural products for an effective treatment for obesity-related diseases, such as cardiometabolic diseases, it seems best to redirect the immunological activity of cells toward the attenuation of immune response, without the potential for unwanted side effects.

Our previous results demonstrated that riboflavin is important for proper macrophage activity. The macrophage RAW 264.7 cell line cultured under conditions of riboflavin deprivation showed reduced proliferation and increased susceptibility to apoptosis. This riboflavin deficiency also affected the cell adhesion, respiratory burst and phagocytosis of macrophages [15], leading to a pathological pro-inflammatory response manifested by excessive TNFα and high-mobility group box 1 (HMGB1) release [16]. On the other hand, riboflavin supplementation seems to be a promising anti-inflammatory strategy which has been repeatedly identified in numerous studies, performed both on in vitro and in vivo models [17,18,19,20]. The anti-inflammatory properties of riboflavin were manifested by the reduction of crucial cytokines such as TNFα, interleukin 1β (IL-1β), IL-6, and HMGB1 as well as inducible nitric oxide synthase (iNOS) and nitric oxide (NO). Considering the impact of riboflavin on macrophage functions, and its important role in the generation of obesity-related inflammation, we assume that the immunological activity of adipocytes may also be dependent on the presence of riboflavin, and riboflavin may affect the intensity of the pro-inflammatory activation of adipocyte-macrophage co-cultures. The present study was designed to assess the modulating effect of riboflavin on the pro-inflammatory activity of adicopocyte-macrophage co-cultures expressed by the production of selected cytokines, adipokines, NO, matrix metalloproteinase-9 (MMP-9) and its inhibitor TIMP-1, as well as macrophage migration to adipocyte-harvested conditioned medium.

## 2. Results

Riboflavin is known to modulate the pro-inflammatory activity of macrophages [15,16,17,18,19,20]. In the present study, the impact of riboflavin supplementation was analyzed with respect to the co-culture of macrophages and adipocytes; both of these exhibited an inflammatory effect.

### 2.1. Riboflavin Decreases Expression and Release of Pro-Inflammatory Cytokines

The production of TNFα, IL-6, IL-1β and MCP-1 pro-inflammatory cytokines was evaluated on two levels: genes and proteins secreted by lipopolysaccharide (LPS)-activated cells in the presence of three high doses of riboflavin (300, 500 and 1000 nM) in relation to the physiological dose of riboflavin (10.4 nM). The gene expression of cytokines was measured by real-time PCR using the ΔΔCt method in mature adipocytes differentiated from the 3T3 L1 cell line and macrophage RAW 264.7 cell line, except for IL-1β analysis which was performed only for RAW 264.7 cells. The release of cytokines by co-cultures of adipocytes and macrophages to cultured medium was determined by enzyme-linked immunosorbent assays (ELISA).

**Figure 1:**
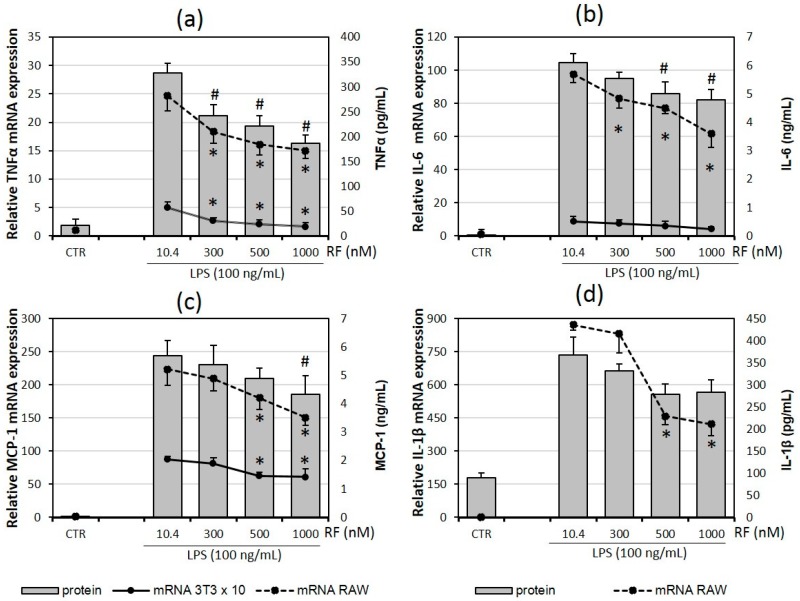
Effects of various riboflavin concentrations (10.4; 300; 500; 1000 nM) on LPS-induced: tumor necrosis factor-alpha (TNFα; **a**), Interleukin 6 (IL-6; **b**), monocyte chemotactic protein 1 (MCP-1; **c**) and interleukin 1 beta (IL-1β; **d**) cytokine mRNA expression and release of cytokines by adipocyte-macrophage co-cultures. Fully differentiated adipocytes 3T3 L1 and macrophages RAW 264.7 were cultured in 12-well dishes with inserts (0.4 μm pore size) for 6 h (mRNA expression; rtPCR) or 24 h (cytokine release; ELISA tests) after LPS stimulation (100 ng/mL). CTR – control group without LPS stimulation. The results are expressed as means + SE of five independent experiments (* *p* < 0.05 significance compared with 10.4 group for mRNAs expression; # *p* < 0.05 significance compared with 10.4 group for cytokines release to supernatant). Statistical significances were determined with the Duncan *t*-test.

The experimental results showed a significant decrease in TNFα mRNA expression in both analyzed cell types in the presence of all used supplementation doses of riboflavin (RF). The reduction of gene expression was accompanied by the attenuation of TNFα secretion by adipocyte-macrophage co-cultures (Figure 1a). A statistically significant diminution was also observed in the case of IL-6 gene expression in RAW 264.7 cells treated by each high dose of riboflavin. In contrast to macrophages, the mRNA level for IL-6 did not show significant changes in 3T3 L1 adipocytes, while the IL-6 secretion by RAW 264.7-3T3 L1 co-culture was lowered in the highest RF concentrations: 500 nM and 1000 nM (Figure 1b). MCP-1 expression and secretion were partially impaired by RF supplementation. We observed a significant reduction of mRNA expression in the two highest doses of RF in both cell types, but MCP-1 secretion was sensitive only for the highest RF concentration (Figure 1c). For the last studied cytokine, we obtained reliable results from gene expression analysis only for the RAW 264.7 cells. The mRNA level for IL-1β decreased in macrophages treated by RF at the concentrations of 500 nM and 1000 nM. The observed reduction of IL-1β secretion in the presence of these RF doses was not statistically significant (Figure 1d). We observed the opposite effect of riboflavin in the case of anti-inflammatory interleukine-10 (IL-10). The gene expression of IL-10 was significantly elevated in the presence of 500 nM and 1000 nM RF compared to the cells treated with a physiological RF concentration. The level of IL-10 secretion was also increased in the mentioned groups.

In summary, our results clearly demonstrated the potential of high doses of riboflavin to lower the pro-inflammatory activities of macrophages and adipocytes expressed as the production and the secretion of TNFα, IL-6, IL-1β and MCP-1 cytokines. The sensitivity threshold for the RF concentration is specific for each cytokine.

### 2.2. Riboflavin Supplementation Impairs Adipokine Expression and Secretion by Adipocytes

**Figure 2:**
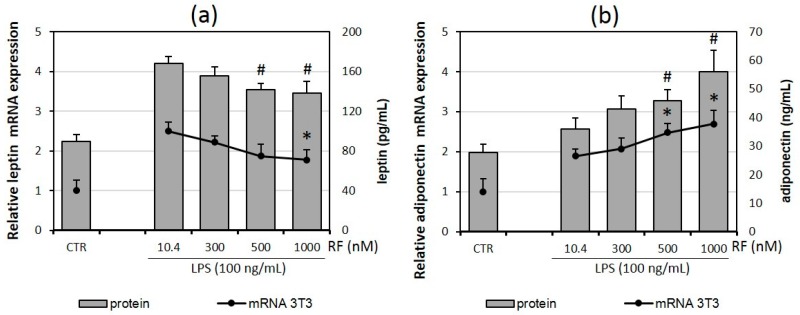
Effects of various riboflavin concentrations (10.4; 300; 500; 1000 nM) on LPS-induced adipokines: leptin (**a**) and adiponectin (**b**) mRNA expression by adipocytes 3T3 L1 and adipokine release by adipocyte-macrophage co-cultures. Fully differentiated adipocytes 3T3 L1 and macrophages RAW 264.7 were cultured in 12-well dishes with inserts (0.4 μm pore size) for 6 h (mRNA expression) or 24 h (cytokine release) after LPS stimulation (100 ng/mL). CTR – control group without LPS stimulation. The results are expressed as means + SE of five independent experiments (* *p* < 0.05 significance compared with 10.4 group for adipokine mRNAs expression; # *p* < 0.05 significance compared with 10.4 group for adipokine release to supernatant). Statistical differences were determined with the Duncan *t*-test.

In the next step of our study, we analyzed two cytokines typical for adipocytes (termed adipokines: leptin and adiponectin) on gene and secreted protein levels. The amount of leptin transcript reduced with increases in the riboflavin doses, but only the highest RF concentration (1000 nM) caused a statistically significant decrease in leptin production (*p* < 0.05), whereas the release of leptin measured in culture medium was negatively impaired in the presence of the two highest RF doses (500 nM and 1000 nM) (Figure 2a).

We observed the opposite effect of riboflavin on adiponectin expression in both mRNA and released protein levels. The adiponectin gene expression was significantly elevated in adipocytes cultured in the presence of 500 nM and 1000 nM RF compared to the cells treated with a physiological RF concentration. The level of adiponectin secretion was also increased in the mentioned groups (Figure 2b).

No effect was observed for resistin levels, which may suggest that riboflavin, at the tested concentrations, is not significantly involved in its expression and/or release.

Considering that adipokines can modulate the synthesis of other cytokines in adipose tissue and given the results presented in Figure 1 and Figure 2, we can conclude that our results support those of previous reports showing that leptin stimulates the production of pro-inflammatory cytokines [21,22], while adiponectin acts as an anti-inflammatory factor [23].

### 2.3. Expression of HMGB1 Is Impaired by High Doses of Riboflavin

**Figure 3:**
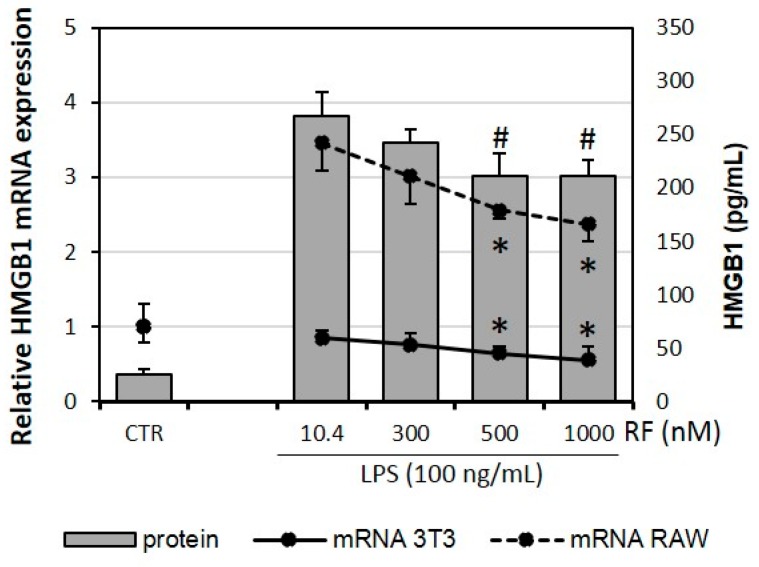
Effects of various riboflavin concentrations (10.4; 300; 500; 1000 nM) on LPS-induced HMGB1 mRNA expression and release by adipocyte-macrophage co-cultures. Fully differentiated adipocytes 3T3 L1 and macrophages RAW 264.7 were cultured in 12-well dishes with inserts (0.4 μm pore size) for 6 h (mRNA expression) or 24 h (HMGB1 release) after LPS stimulation (100 ng/mL). CTR – control group without LPS stimulation. The results are expressed as means + SE of five independent experiments (* *p* < 0.05 significance compared with 10.4 group for HMGB1 mRNA expression; # *p* < 0.05 significance compared with 10.4 group for HMGB1 release to supernatant). Statistical differences were determined with the Duncan *t*-test.

Previous studies report the ability of high-mobility group box 1 (HMGB1) to induce the pro-inflammatory activation of macrophages [24] and the regulatory effect of riboflavin on its macrophage activity in the RAW 264.7 cell line [16]. The present analysis confirmed the ability of RF to reduce the mRNA level of HMGB1 in RAW 264.7 cells. We also found an effect of the attenuation of HMGB1 expression in the 3T3 L1 adipocyte cell line. In both cases, a statistically significant reduction was achieved in the presence of the highest concentration of RF (500 nM and 1000 nM). The impact of RF on HMGB1 secretion in adipocyte-macrophage co-culture corresponded to its gene expression (Figure 3).

### 2.4. Macrophage Migration Is Reduced in the Presence of High Doses of Riboflavin

**Figure 4:**
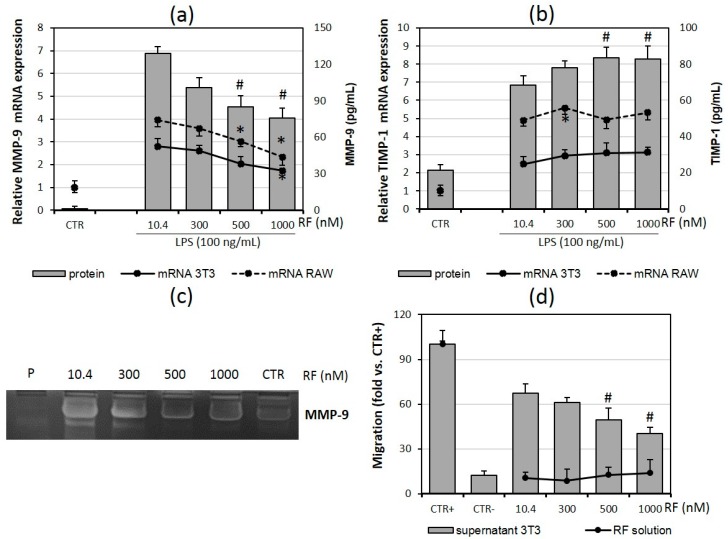
Effects of various riboflavin concentrations (10.4; 300; 500; 1000 nM) on LPS-induced metalloproteinase-9 (MMP-9; **a**,**c**) and its inhibitor TIMP-1 (**b**) mRNA expression and release by adipocyte-macrophage co-cultures. Fully differentiated adipocytes 3T3 L1 and macrophages RAW 264.7 were cultured in 12-well dishes with inserts (0.4 μm pore size) for 6 h (mRNA expression) or 24 h (cytokine release) after LPS stimulation (100 ng/mL). CTR − control group without LPS stimulation. (**d**) Macrophages RAW 264.7 migration to supernatants from adipocytes cultured in various riboflavin concentrations for 24 h and migration to various riboflavin concentrations (RF solution). Normal medium was used as a negative control (CTR−), fMLP was used as a positive control (CTR+). The results are expressed as means + SE of four independent experiments (* *p* < 0.05 significance compared with 10.4 group for mRNAs expression; # *p* < 0.05 significance compared with 10.4 group for MMP-9 activity, TIMP-1 release and migration to supernatant). Statistical significances were determined with the Duncan *t*-test.

Since adipokines secreted by adipocytes act as chemoattractants for macrophages and this leads to the infiltration of adipose tissue [25]; the next stage of our study was designed to assess the influence of riboflavin on macrophage chemotaxis (Figure 4). The results showed that the highest doses of RF (500 nM and 1000 nM) reduced RAW 264.7 migration towards chemoattractants present in supernatant collected from cultures of 3T3 L1 adipocytes (Figure 4d).

Cell migration is promoted by the proteolytic activity of matrix metalloproteinases (MMPs) and controlled by tissue inhibitors of metalloproteinases (TIMPs) [26]; therefore, we also analyzed the effect of riboflavin on MMP-9 and TIPM-1 expression and secretion. MMP-9 expression in adipocytes and macrophages and secretion in co-cultures was reduced under the influence of the two highest concentrations of RF (Figure 4a). This was accompanied by the significant enhancement of TIMP-1 secretion, but the observed increase in TIMP-1 expression on the mRNA level was not statistically important in the variants with the highest RF doses (Figure 4b).

Zymography was performed to determine MMP-9 action and it revealed that the activity of this enzyme was reduced in the two highest RF concentrations (Figure 4c) and the activity reduction was proportional to the mRNA level of MMP-9 (Figure 4a).

### 2.5. Riboflavin Affects Nitric Oxide Production

The role of nitric oxide (NO) and an inducible nitric oxide synthase (iNOS) is well characterized in the immune response [27].

**Figure 5:**
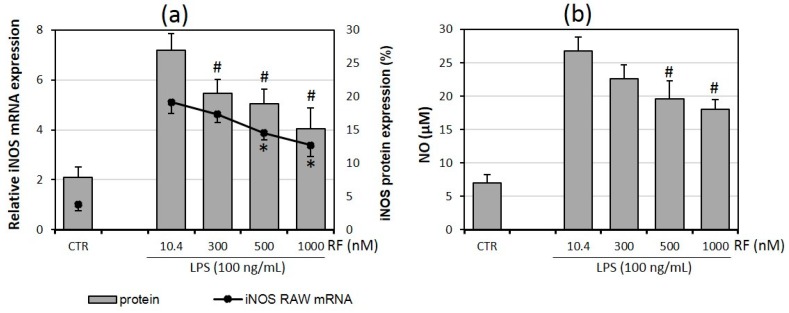
Effects of various riboflavin concentrations (10.4; 300; 500; 1000 nM) on LPS-induced nitric oxide synthase (iNOS) mRNA and protein expression (**a**) and nitric oxide release (NO; **b**) by RAW 264.7 macrophages cultured in adipocyte-macrophage co-cultures. Fully differentiated adipocytes 3T3 L1 and macrophages RAW 264.7 were cultured in 12-well dishes with inserts (0.4 μm pore size) for 6 h (mRNA, protein expression) or 24 h (NO release) after LPS stimulation (100 ng/mL). CTR – control group without LPS stimulation. The results are expressed as means + SE of five independent experiments (* *p* < 0.05 significance compared with 10.4 group for iNOS mRNA expression; # *p* < 0.05 significance compared with 10.4 group for iNOS protein expression and NO release to supernatant). Statistical differences were determined with the Duncan *t*-test.

The results obtained in the present study revealed that the gene and protein expression of iNOS in RAW 264.7 macrophages was significantly impaired by riboflavin supplementation (Figure 5a). This was correlated with the decreased level of the NO quantity in supernatants from adipocyte-macrophage co-cultures (Figure 5b).

### 2.6. Phosphorylation of NFκB Is Lowered in Riboflavin-Treated Adipocytes and Macrophages

**Figure 6:**
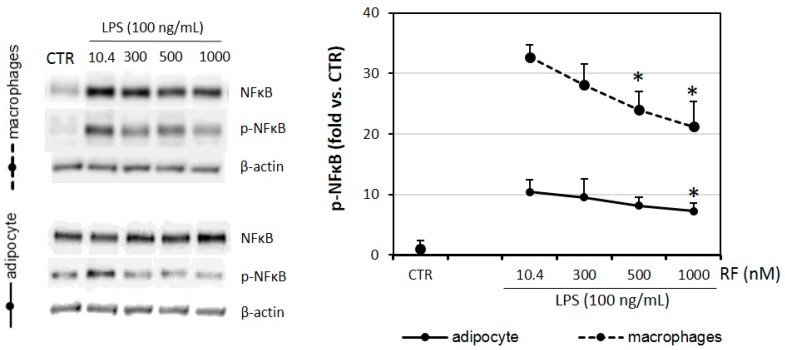
Effects of various riboflavin concentrations (10.4; 300; 500; 1000 nM) on LPS-induced phosphorylation of nuclear factor κB (p-NFκB) in co-cultured RAW 264.7 macrophages and 3T3 L1 adipocytes. Fully differentiated adipocytes and macrophages were cultured in 12-well dishes with inserts (0.4 μm pore size) upon LPS stimulation (100 ng/mL). CTR – control group without LPS stimulation. The results are expressed as means + SE of five independent experiments (* *p* < 0.05 significance compared with 10.4 group). Statistical differences were determined with the Duncan *t*-test.

Nuclear factor kappa B (NFκB) is an important activator of macrophage [28] and adipocyte [29] inflammatory responses. Therefore, the last step of our studies was planned to determine the phosphorylation of NFκB in the presence of riboflavin supplementation. The results showed a decrease in NFκB activation in both macrophages and adipocytes treated with the higher concentrations of RF (500 nM and 1000 nM for RAW 264.7 cells, 1000 nM for 3T3 L1 adipocyte cells) and a more significant effect of the reduction of NFκB phosphorylation was found in RAW 264.7 macrophages (Figure 6).

The reduction of NFκB phosphorylation was understandable in the light of previous results showing decreases of pro-inflammatory cytokines (Figure 1 and Figure 2a).

### 2.7. Viability of Adipocyte-Macrophage Co-Culture

Evaluation of cells’ viability as well as the percentage of apoptotic and necrotic cells has shown no significant effect of riboflavin administration on the parameters mentioned above (data not shown).

## 3. Discussion

Riboflavin (vitamin B2), as a precursor for flavin mononucleotide (FMN) and flavin adenine dinucleotide (FAD) synthesis, is required for proper functioning of numerous enzymes, especially from the oxido-reductase group [14,30,31]. Due to the nature of building cofactors, riboflavin is indispensable for energy generation by aerobic cells, mitochondrial metabolism and fatty acid oxidation (for a review see [30,31]). For these reasons, it determines the proper operation of numerous systems of the body, in particular the nervous, cardiovascular, endocrine and immune systems [14,30]. As we presented in our previous study [17], riboflavin supplementation significantly alters the effector function of macrophages stimulated with both bacterial-derived LPS as well as yeast-derived zymosan, leading to a reduction of their pro-inflammatory activity. High pharmacological doses of riboflavin effectively inhibit the HMGB1 release, an important factor in the pathogenesis of endotoxemia [16], and offer a promising therapeutic strategy in sepsis and septic shock as a result of attenuated inflammation, consequently leading to an increase in survival rates among septic animals [32,33,34]. Given the significant effects of riboflavin supplementation in the creation of an anti-inflammatory microenvironment by macrophages, an essential component of the fat tissue, the current study demonstrates the impact of this condition on the adipocyte-macrophage interaction in the context of moderate inflammation, which is associated with the state of obesity. This analysis appears to be particularly important because of the essential role of obesity-related inflammation in the development of numerous diseases and syndromes associated with obesity, such as an insulin resistance, diabetes mellitus 2 or arteriosclerosis.

The present study clearly demonstrates that riboflavin is an essential micronutrient for the proper functioning of fat cells, and its supplementation leads to the induction of promising attenuation in the pro-inflammatory activation of adipocyte-macrophage co-cultures. Given that the excess riboflavin supplied into the organism is quickly removed by renal secretion, and the fact that it is not stored, actually overdose symptoms are not described [14,35]. The riboflavin concentrations evaluated in the current study do not exert any cytotoxic effect on adipocyte-macrophage co-cultures, which was confirmed by an apoptosis/necrosis study. Moreover, the state of riboflavin supplementation was accompanied by a marked decline in the key inflammatory factors, such as NFκB phosphorylation or TNFα, IL-6, IL-1β, HMGB1, NO and leptin expression and release. These observations may suggest that riboflavin supplementation leads to the creation of a favorable environment for protection against the development of insulin resistance and other obesity-related disorders.

Obesity is a state characterized by a massive infiltration of adipose tissue by macrophages, the activity of which contributes to insulin resistance by the excessive release of pro-inflammatory cytokines such as TNFα or IL-6 [2]. Together with adipocytes, macrophages create crosstalk between inflammation and insulin resistance [30]. As reported by Lumeng et al. [36], macrophage polarization in adipose tissue differs depending on the tissue type and adiposity. In adipose tissue of lean individuals M2 macrophages predominate, whereas in fat tissue from obese people M1 macrophages predominate. Macrophage infiltration is proportional to fat mass, and in obese fat tissue the number of infiltrating macrophages is significantly higher than in lean tissue. MCP-1 chemokine, released by both adipocytes and macrophages from fat tissue, is responsible mainly for macrophages’ influx to the adipose tissue [25]. Our study indicated that riboflavin supplementation reduces the whole level of chemoattractants released by adipocytes, and consequently reduces the intensity of macrophage migration.

The impact of riboflavin on cytokine expression and release is evidenced by a significant reduction in the TNFα level, considered to be one of the main cytokines related to the development of insulin resistance. TNFα inhibits the peroxisome proliferator–activated receptor gamma (PPAR-γ), a regulator of glucose metabolism essential for insulin sensitivity [37]. Through enhancement of adiponectin and inhibition of TNFα release, PPAR-γ improves the insulin resistance state in obese subjects [38]. As reported by Sartipi et al. [30], MCP-1 overexpression in obese fat tissue also impairs adipocyte insulin sensitivity. The overexpression of crucial pro-inflammatory cytokines, e.g., TNFα, IL-6 and MCP-1, which is associated with the state of obesity, is closely connected to insulin resistance as well as diabetes mellitus 2 or cardiovascular syndrome development [39,40]. Our data suggest that riboflavin supplementation, by attenuation of the pro-inflammatory activation of adipocyte-macrophage fat tissue components, may reduce the likelihood of obesity-related disease development, such as insulin resistance or diabetes mellitus 2.

Obesity-related elevation of TNFα may also be connected with the development of hyperleptinemia in obese subjects, because of leptin stimulation by TNFα [41,42]. As presented by Zhou and Rui [43], leptin is a key mediator in adipose tissue–brain communication and is important in the maintenance of energy homeostasis and normal body weight. The lack of the leptin gene leads to a lethal form of obesity development [44] and an alteration in leptin pathway homeostasis, and the abnormally high leptin level observed in obese subjects could result in leptin resistance because of decreased leptin receptor signaling (for a review, see [43]). In our study, the level of both leptin expression and release was significantly decreased in riboflavin-supplemented adipocytes compared with adipocytes cultured in the physiological riboflavin concentration. Moreover, the leptin level was significantly higher after LPS stimulation, which remains in contradiction to Finck et al.’s [45] study that has indicated no effect of LPS stimulation on increasing leptin secretion in adipocyte culture. This difference could be, at least in part, explained by using various experimental models. The results published by Finck et al. were obtained for the primary adipocytes, while the current study assesses the interaction in adipocyte-macrophage co-cultures. As we suspect, the elevation in the leptin level in adipocytes co-cultured with LPS-sensitive macrophages could be a result of excessive TNFα release by the macrophages. The effect of increased leptin release by adipocytes after stimulation with TNFα was also observed in Finck et al.’s studies. Nevertheless, with regard to Kirchgessner et al.’s studies [41] indicating a post-translational mechanism to control leptin release due to TNFα stimulation, we can assume that the observed decrease in the expression and release of leptin upon riboflavin supplementation may be only partially explained by the concomitant reduction in TNFα. Further studies are needed to determine the mechanism of leptin inhibition by riboflavin. Nevertheless, our results suggest that riboflavin supplementation could prevent hyperleptinemia in obese subjects.

Adiponectin, an adipokine also known as adipocyte complement-related protein (Acrp30), is important in lipid metabolism, insulin sensitization and anti-inflammatory response [23]. The serum level of adiponectin, in contrast to leptin, is reduced in the state of obesity, insulin resistance and diabetes mellitus and its reduction plays a significant role in their development [46,47]. Adiponectin has both anti-inflammatory and anti-diabetic properties and its action is closely connected with the attenuation of obesity-related inflammation [48,49]. As reported by Lira et al. [50], adiponectin participates in the inhibition of NFκB, a crucial transcription factor of the pro-inflammatory pathway, and moreover reduces HMGB1 release by LPS-stimulated macrophages [51]. As reported by Shimizu et al. [52], attenuation of HMGB1 by adiponectin also occurs in adipocytes as a result of the inhibition of JNK signaling. HMGB1 is a DNA-binding protein located in quiescent cells in their nuclei which stabilize nucleosomes and participate in replication, recombination and transcription processes [51,52]. Nevertheless, upon activation or cell death, HMGB1 can also be released from cells through active or passive mechanisms [53]. In extracellular milieus, HMGB1 acts as a strong pro-inflammatory cytokine that leads to cell activation through the interaction with Toll-like receptor 4 (TLR4) or the receptor for advanced glycation end product (RAGE) [51,52,53]. HMGB1 has been implicated in those mechanisms leading to the development of states such as sepsis or septic shock, wherein it is responsible for the intensification of excessive pro-inflammatory activation of immune cells, as well as in atherosclerosis or rheumatoid arthritis, where elevated levels of HMGB1 have also been observed [53]. Our studies have shown that adipocytes cultured in a riboflavin-enriched environment produce significantly more adiponectin than adipocytes from the control group. This result suggests that riboflavin could reduce obesity-related hypoadiponectinemia and, at least in part, explain lower pro-inflammatory activation and reduced release of TNFα, IL-6 and HMGB1 as well as NFκB phosphorylation because of the improvement of the action of adiponectin, an important inflammation controller [23]. The anti-inflammatory activity of adiponectin is also associated with the induction of the expression and release of anti-inflammatory factors such as IL-10 or IL-1RA in leukocytes [54]. Our study has shown that IL-10 is significantly higher in co-cultures maintained in a riboflavin-enriched environment, and this could be, at least in part, connected with higher adiponectin levels, which in turn induce IL-10 release [53]. Moreover, Lumeng et al. [36] mentioned that IL-10 protects against TNFα-induced insulin resistance in adipocytes, and in turn reduces TNFα levels in macrophages through STAT3 activation [55] and in adipocytes through activation of PI3K pathways via insulin receptor substrate (IRS) protein [56]. Moreover, a reduction in TNFα and other pro-inflammatory cytokines could be in part a result of NFκB inhibition via adiponectin overexpression [56].

In conclusion, the results presented in this paper suggest that riboflavin supplementation induces functional changes in adipocyte-macrophage co-cultures and leads to a reduction in the intensity of their pro-inflammatory, pro-insulin resistance as well as their pro-diabetes activity. Through a decrease in pro-inflammatory cytokines, riboflavin supplementation may reduce the likelihood of the development of obesity-associated hyperleptinemia and hypoadiponectinemia, and thus decrease the risk of diseases associated with obesity.

## 4. Materials and Methods

### 4.1. Chemicals and Materials

DMEM medium, antibiotics: streptomycin and penicillin, fetal bovine serum (FBS) were purchased from PAA (Pasching, Austria). Riboflavin, lipopolysaccharide (LPS), 2′,7′-dichlorodihydrofluorescein diacetate (DCFDA), β-actin antibody were from Sigma-Aldrich (St. Louis, MO, USA). Annexin V kit’s and Cytofix/Cytoperm solution were purchased from BD Biosciences Pharmingen (San Diego, CA, USA). The RNeasy Plus Mini Kit (74134) for RNA isolation was obtained from Qiagen (Hilden, Germany). The High Capacity RNA-to-cDNA Kit (4387406) and TaqMan Gene Expression Master Mix (4369026) and primers were purchased from Applied Biosystems (Foster City, CA, USA) and mouse adiponectin, resistin, and TIMP-1 ELISA kits from NOVEX (ThermoFisher; Foster City, CA, USA). Phospho-NFκBp65 and total NFκBp65 and secondary antibodies were from Cell Signaling Technology (Beverly, MA, USA). ELISA kits for TNFα, IL-6, HMGB1 and leptin were purchased from IBL International (Hamburg, Germany). ELISA kits for mouse MCP-1, IL-1β, IL-10, and TGFβ were purchased from R&D BioVendor (Heidelberg, Germany).

### 4.2. Cell Culture and Experimental Design

The study was conducted on a *Mycoplasma*-free mouse adipocytes (differentiated from the pre-adipocytes 3T3 L1 cell line) and mouse RAW 264.7 macrophages. The RAW 264.7 cell line was purchased from the European Type Culture Collection (ETCC, Sigma) and 3T3 L1 pre-adipocytes were kindly provided by Professor Alicja Józkowicz from the Department of Medical Biology, Jagiellonian University. Cells were incubated in customized (riboflavin-depleted) high-glucose DMEM medium supplemented with riboflavin to achieve the required riboflavin concentration (10.4; 300; 500 or 1000 nM). For the differentiation of pre-adipocytes to adipocytes, cells were grown until confluence in DMEM medium supplemented with 1% antibiotic solution (streptomycin/penicillin) and 10% calf serum. Two day after cell reached confluency, the growth medium was replaced on differentiation medium for the next two days (DMEM medium with 10% fetal bovine serum, 0.5 mM isobutylmethylxanthine – IBMX, 0.25 μM dexamethasone). Thereafter, the cells were incubated in a DMEM medium containing 10% FBS and 1 μg/mL insulin to day 10. Oil-Red-O staining was used for evaluation of adipocyte differentiation and lipid droplets deposition. For the co-culture study, fully-differentiated adipocytes were maintained in 12-well plates with RAW 264.7 cells cultured in plate inserts (0.4 μm pore size). For induction of the state of mild inflammation cells, co-cultures were stimulated with lipopolysaccharide (LPS; 100 ng/mL; *Escherichia coli*, serotype 0111: B4) for 6 (gene expression studies) or 24 h (protein expression and release studies). During all procedures, cells were cultured under standard conditions (37 °C, 5% CO_2_). Fresh cells were used for cytometric analyzes and RNA isolation. Supernatants and cell pellets were frozen (−60 °C) for future quantification of protein and nitric oxide levels.

### 4.3. Determination of Apoptosis

Cell viability and the percentage of apoptotic and necrotic cells were determined after 24 h co-culture using a commercial Annexin V kit according to the manufacturer’s instructions. Analyzes were performed on a FACScan cytometer (FACSCalibur™; BD Biosciences, San Diego, CA, USA) using CellQuest software (Becton Dickinson, San Diego, CA, USA). For the analysis of data, 10,000 events were acquired.

### 4.4. Quantitative Real-Time PCR Assay

Total cell RNA from adipocytes and macrophages was extracted using an RNeasy Plus Mini Kit according to the manufacturer’s instructions. The concentration and quality of RNA were measured using a NanoDrop 2000 spectrophotometer (Thermo Scientific, Waltham, MA, USA). cDNA was synthesized with oligo(dT) primer (TNF-α Assay ID: Mm00443260_g1, IL-6 Assay ID: Mn00446190_m1, MCP-1 Assay ID: Mm00441242_m1, IL-1β Assay ID: Mm00434228_m1, HMGB1 Assay ID: Mm00805422_m1, iNOS Assay ID: Mm00440502_m1, TGFβ Assay ID: Mm01178820_m1 MMP-9 Assay ID: Mm00442991_m1, TIMP-1 Assay ID: Mm01341361_m1, adiponectin Assay ID: Mm00456425_m1, leptin Mm00434759_m1, resistin Assay ID: Mm00445641_m1) using a High Capacity RNA-to-cDNA Kit in thermoblock Thermostat plus (Eppendorf) according to the manufacturer’s protocol. Gene expressions were evaluated using TaqMan Gene Expression Master Mix in the StepOne Plus system (Applied Biosystems). The expressions of analyzed genes were normalized to endogenous GAPDH gene expression (glyceraldehyde phosphate dehydrogenase; Assay ID: Mm99999915_g1) selected as the housekeeping gene. The relative expression of each gene (RQ) was calculated using the 2^−ΔΔCt^ method.

### 4.5. Western Blotting Assay

Total proteins of NFκB as well as its phosphorylated form were extracted from adipocyte pellets according to the manufacturer’s protocol. The samples were normalized to the protein content after evaluation with the BCA protein assay kit. The normalized protein samples were subjected to the SDS-PAGE gel (10%–15%), and after electrophoretic separation were transferred onto a PVDF membrane. The non-specific binding sites were blocked with 5% dried milk in TBSS for 1 h (37 °C). Then, membranes were incubated overnight with primary antibodies (4 °C; 1:1000), and then with secondary antibodies conjugated with horseradish peroxidase (HRP; 1:2000) for 2 hours at room temperature. After reaction with DAB, membranes were imaged and the relative protein expressions were normalized to the internal references expression (β-actin).

### 4.6. Examination of Cytokine and Adipokine Release

After 24 h incubation with or without LPS, the supernatants were collected and the levels of cytokines were quantified using a commercial Elisa Kit. The levels of TNFα, IL-6, HMGB1, MCP-1, IL-1β, IL-10, TGFβ, TIMP-1, adiponectin, resistin and leptin were determined according to the manufacturer’s instructions and measured on a spectrophotometer (Expert Plus, ASYS/Hitech, Eugendorf, Austria).

### 4.7. Analysis of iNOS Protein Expression and NO Release

The level of inducible nitric oxide synthase (iNOS; NOS2) was detected by flow cytometry after labeling with a fluorescent antibody. Briefly, after a 24 h co-culture RAW 264.7 cells were detached, blocked with Fc-block (0.5 mg/mL; 1:200; 20 min; 4 °C), fixed, and permeabilized with Cytofix/Cytoperm solution according to the manufacturer’s instructions. Afterwards, cells were incubated with rabbit polyclonal IgG (0.2 mg/mL; 1:100; 20 min; 4 °C) and goat anti-rabbit-phycoerythrin antibodies were used (0.2 mg/mL; 1:100; 20 min; 4 °C). For data analysis, 10,000 events were collected on an FACScan cytometer (FACSCalibur™; BD Biosciences) using a CellQuest software (Becton Dickinson). Determination of nitric oxide (NO) levels in supernatants from co-culture was performed after 24 h stimulation with LPS using an Arrowstraight™ Nitric Oxide measurement system (Lazar Research Laboratories, Los Angeles, CA, USA), as previously described by Magierowska et al. [57].

### 4.8. Determination of MMP-9 Activity Using the Zymography Technique

Gelatinolytic activity of pro- and active forms of metalloproteinase-9 (MMP-9) in the supernatant from co-cultures of adipocytes and macrophages was evaluated after 24 h stimulation with LPS by gelatin zymography, as previously described by Mazur et al. [20].

### 4.9. Macrophages Migration

To assess the migration of macrophages, a 48-well microchemotaxis chamber (Neuro Probe) was used. The test was performed according to the manufacturer’s protocol. Briefly, the lower wells of the chamber were filled with adipocyte supernatants collected 24 h after LPS stimulation, control supernatants, fMLP as a positive control, or DMEM medium as a negative control. The lower wells of the chamber were covered with a polycarbonate membrane (3 μm pore size; Neuro Probe). The wells in the upper chamber were filled with 50 μL of macrophages RAW 264.7 (2 mln/mL) and incubated for 45 min in standard culture conditions. After, the membrane was washed in PBS and stained with Diff-Quick solution (Medion Diagnostic). The macrophages that migrated to the lower side of the membrane were counted in four microscopic fields in each wells. The results are expressed as the mean numbers of migratory macrophages per well.

### 4.10. Statistical Analysis

Data were tested for normality of distribution, and differences among groups were determined using the Duncan′s new multiple range test 3.1. All data were expressed as means ± standard error (X ± SE) with the level of statistical significance (*p*) set at 0.05.
